# Dr. Ferguson, on Leeches

**Published:** 1805-04-01

**Authors:** Andrew Ferguson


### *To the Editors of the Medical and Physical Journal.*

Gentlemen,

Every practitioner must be sensible of the utility of local blood-letting in several inflammatory affections, which cannot with safety be performed but by leeches. It therefore gave me great pleasure to find some of your correspondents, in the Medical and Physical Journal, making their preservation and management a subject of inquiry. Mr. Wilkinson has pretty fully explained what relates to their preservation, management and application: we may, however, expect a good deal of information, with respect to their natural history, from some of your correspondents who reside upon the spot where the leeches in general use are obtained, which is in some part of Norfolk. I recollect, that about eight or nine years ago, leeches were very plenty in Aberdeen; people came from Musselburgh and Edinburgh, hawking them through the apothecaries shops here. They were sold from nine to ten shillings the hundred. They generally had several thousands of them, which they carried in a small cask, or firkin, without any water. They washed them, however, with pure spring water twice every day. When I looked in among them, they appeared all in motion, and were very clean, and free from those slimy skins in the form of small rings, which leeches generally cast off every other day. Their continual motion facilitates this, and helps to keep them clean. From their healthy state with this treatment, I should think that leeches ought to be kept in a large capacious vessel, and when they are cleaned once every day, should have but a small quantity of water round them.

I once bought a hundred leeches from a hawker, which almost all died soon after. When I first got them, they had not that vivacity and strength of motion which sound and healthy leeches have; they were soft and flabby, and did not recoil into a firm knot (when taken up in the hand and pressed a little), which they generally do when in perfect health.

I gave them fresh water every day, and freed them from all slime and filth, yet the water was bloody every morning, and some of them dead. Those that died were quite flabby, their mouths swelled and white, and sometimes several hard knots in different parts of their bodies; but most frequently towards their neck. Upon dissection, these these tumours appeared to be owing to black coagulated blood. When the mortality was so great among them, I added a little lump sugar to the water; and, after that, I tried a little peat turf; but nothing that I could think of was of any service in restoring them to health. I found that putting them in the warm rays of the sun gave them a little fresh vigour. As leeches at that time were easy to be had, I was not very anxious of trying experiments with them. It is of the utmost importance to have leeches strong and healthy; the first that are caught, or what are called spring leeches (I am told) are generally stronger than those that are caught in the summer. But very frequently good leeches, after keeping them for some time in quantities together, will turn sickly and die. When this mortality is beginning, the best prophylactic is to separate them into small parties. The first sign of an approaching mortality among leeches, is the water turning bloody: when this appears, they should be instantly separated, kept in small numbers, and supplied regularly once every day with fresh water. From my own observations, I have found that pure spring water, which has run in a brook where it could not be impregnated with metalic substances, is best for keeping them. The water of the upper course of the city of Aberdeen, is much better for preserving leeches than the lower course, where the water is immediately conveyed from the spring; the former is softer, owing to its being exposed to the action of the atmosphere, which deprives it in some degree of its fixed air. Leeches are likewise naturally to be found in brooks where the same water runs, which is a sign of its being more agreeable to them.

I believe, that where leeches are kept in quantities together, the water should be renewed daily, and all the slime and filth well washed away from them; but where a few are only kept in a phial, it is not so absolutely necessary. I have kept six dozen of them together, and not changed the water more than once in three days during the cold weather of winter: but, in summer, it is necessary that they should have fresh water oftener. I have, during cold weather, added water to them almost at the freezing point, without doing them any mischief.

It is also necessary that they should be kept in a well aired place, free from smoke and the effluvia of drugs. An apothecary's shop is one of the worst places for keeping leeches. Leeches are peculiarly delicate, and a very little injures them. Noise of every kind proves hurtful to them them, but especially a thunder storm; they are always unwell after it, and very commonly a mortality commences soon after. An apothecary, when he intends to handle leeches, ought always to have his hands clean washed; for the smallest particle of some drugs adhering to the hands, has been known to occasion a mortality amongst them. Motherby, in his Medical Dictionary, observes, that Themison is the first who takes notice of leeches, and refers the curious to the following writers on this subject, viz. Aldrovandus, Gesner, Botallus, Sebezius, Heurnius, Cransius, Schroder, and Stahl. He says, those should be chosen whose backs are striped and bellies spotted, and which are taken from running waters that are clear, and have a sandy bed. He orders the water in which they are kept to be renewed every three or four days at the latest, and a little sugar may be added to the water in which they are kept. The quantity of blood which leeches draw, is always in proportion to their size. A large leech generally draws one ounce of blood; but, by applying cloths wrung out of warm water to the orifice, and encouraging it to bleed, double that quantity may be obtained. But sometimes the orifice will bleed a very great quantity when the wound is on a vascular part. I have been called several times to stop the bleeding of a leech, which I could only effect by a good deal of pressure. The wound is of a triangular shape, and of a lacerated nature; an inflammation attends it for several days, while a small black scab forms upon the orifice, which comes off in eight or nine days. Salt is the article most generally used for making leeches vomit the blood they have sucked; but there is another method of emptying them of blood, which I have seen practised, and that is by drawing them through the fingers, and thereby forcing the blood out. This is called striping leeches; a method which, I believe, is used by the catchers of them at some particular seasons. When a leech is full of blood, all those rings upon their surface are perfectly stretched, and they resemble a cylinder filled with blood. A large leech will sometimes suck so long, and be so distended, that in undergoing the vomiting process it will generally expire; therefore a large leech should never be allowed to continue to suck to that degree, as to exhaust her strength by over distention.

There is a species of leech to be found near Aberdeen, of a large size, and yellow colour. Their backs are of a brownish yellow, and their bellies of a lighter yellow; they are only to be met with in the summer; they are known by the the name of horse leeches; people are very much afraid of them when they are barefooted in brooks, where they are to be seen. They imagine, that were they to fix upon them, they would suck all their blood out before they could be removed. These notions have prevented their being applied to any use. One time, when there was a scarcity of leeches, a man brought me half a dozen in a phial, and wished me to apply them to his leg. I was glad of this opportunity of making trial of them; but after using every method I was acquainted with, I could not succeed in making them bite. I even removed part of the cuticle, and tried them upon it; but after several attempts they would not fasten. I then put them into a closet, and did not look at them for several months; I found five of them living, but was much surprized to find one of them gone; because the mouth of the phial was properly secured with a bit of linen rag. In about six weeks after, I missed another; some time afterwards, the third was gone, and then the fourth; which raised my curiosity, and caused me to look rather oftener. I perceived one day, that the two which remained were connected together; one of them appeared dead, and the other living; I looked frequently to see what this phænomenon would end in. The dead leech gradually disappeared, and there remained only one. He continued to live for some time, but appeared to lose his strength gradually, and to turn smaller; at last he died. During the whole time I kept them, which was rather more than a twelvemonth, I only renewed the water twice or three times; yet the water continued pure, sparkling, and free from smell—a small quantity of greenish matter gathered round the sides of the phial. Although my first attempt to make these animals bite proved unsuccessful; yet, as they differ in nothing from those which we get from England but the colour, I am not without hope, but by repeated trials, I may succeed in making them bite, could the prejudices of the people be removed. When they can be had so easily, and appear to be so hardy, could they be employed as a substitute for the other kind, it would not only save a great expence, but also afford us a resource upon all occasions.

When the season arrives that they make their appearance, I shall send a few of them to Mr. Wilkinson for the purpose of making experiments with them.

While writing the above, Feb. 12, 1805, I was sent for to a patient at a place called King's Wells, four miles from Aberdeen,

Aberdeen, affected with Ascites. They called her Isabel Bisset, an unmarried woman, aged 24. The swelling was so very large that she could not turn on her back, but always lay on her side. Her strength appeared to be almost exhausted, and her pulse was scarcely perceivable: although she was at times very sick and faintish, yet she had never fainted altogether. I have seen a good many cases of ascites, but never any accompanied with such an extraordinary abdominal swelling. It is nearly two years since the swelling was first perceived: she complained then of pain in her right side, accompanied with irregular and copious menstruation. Six months ago, she was seized with vomiting of her food and drink, which continued for three months; her belly increased in size every day; her menses disappeared; her thirst increased, and her urine turned more scanty. She has a stool every day, but is incapable of turning herself in bed; and, when she attempts it, her breath goes almost entirely away; she cannot bear to lie upon her back for one minute. Finding it impossible, in her present condition, to be sent to the hospital, I thought it might be a means of alleviating her distress, to perform the operation of paracentesis. With this intention, I went next day with an assistant, and carried along with me a bandage for compressing the abdomen, somewhat resembling Dr. Monro's; but, instead of leather, it was made of strong unbleached linen, lined with flannel, and strong straps for tying. Her circumference, before I put on the bandage, measured four feet and a half. I marked the place where I intended to introduce the trocar, and cut an opening in the bandage opposite it, which could be opened or shut at pleasure, as it was fastened with pins. During the operation, she was not in the least inclined to faint, but kept her spirits very well, and desired the assistants not to withdraw the pressure, otherwise she could not breath, I drew off from her thirteen Scots pints of water, of a tea-green colour, which is equal to forty-eight English pints. Eight days after she was continuing easier, and feels herself very light, but has not strength to sit up, or much appetite for food.

I am, &c.

ANDREW FERGUSON.

Some

